# Trends in Exercise-Related Internet Search Keywords by Sex, Age, and Lifestyle: Infodemiological Study

**DOI:** 10.2196/59395

**Published:** 2024-11-11

**Authors:** Kosuke Uemura, Taiju Miyagami, Mizue Saita, Takuro Uchida, Shun Yuasa, Keita Kondo, Shun Miura, Mizuki Matsushita, Yuka Shirai, Richard Baku Misawa, Toshio Naito

**Affiliations:** 1 Department of General Medicine Faculty of Medicine Juntendo University Tokyo Japan

**Keywords:** exercise prescriptions, sex, age, lifestyle, internet search keywords, infodemiology, demographic, physical activity

## Abstract

**Background:**

Exercise prescription by physicians is beneficial for initiating or intensifying physical activity. However, providing specific exercise prescriptions is challenging; therefore, few physicians prescribe exercise.

**Objective:**

This infodemiological study aimed to understand trends in exercise-related internet search keywords based on sex, age, and environmental factors to help doctors prescribe exercise more easily.

**Methods:**

Search keyword volume was collected from Yahoo! JAPAN for 2022. Ten exercise-related terms were analyzed to assess exercise interest. Total search activities were analyzed by sex and age. Characteristic scores were based on the Japanese prefecture. By performing hierarchical cluster analysis, regional features were examined, and Kruskal-Wallis tests were used to assess relationships with population and industry data.

**Results:**

The top-searched term was “Pilates” (266,000 queries). Male individuals showed higher interest in activities such as “running” (25,400/40,700, 62.4%), “muscle training” (65,800/111,000, 59.3%), and “hiking” (23,400/40,400, 57.9%) than female individuals. Female individuals exhibited higher interest in “Pilates” (199,000/266,000, 74.8%), “yoga” (86,200/117,000, 73.7%), and “tai chi” (45,300/65,900, 68.7%) than male individuals. Based on age, search activity was highest in the 40-49 years age group for both male and female individuals across most terms. For male individuals, 7 of the 10 searched terms’ volume peaked for those in their 40s; “stretch” was most popular among those in their 50s; and “tai chi” and “radio calisthenics” had the highest search volume for those in their 70s. Female individuals in their 40s led the search volume for 9 of the 10 terms, with the exception of “tai chi,” which peaked for those in their 70s. Hierarchical cluster analysis using a characteristic score as a variable classified prefectures into 4 clusters. The characteristics of these clusters were as follows: cluster 1 had the largest population and a thriving tertiary industry, and individuals tended to search for Pilates and yoga. Following cluster 1, cluster 2, with its substantial population, had a thriving secondary industry, with searches for radio calisthenics and exercise bike. Cluster 4 had a small population, a thriving primary industry, and the lowest search volume for any term. Cluster 3 had a similar population to that of cluster 4 but had a larger secondary industry.

**Conclusions:**

Male individuals show more interest in individual activities, such as running, whereas female individuals are interested in group activities, such as Pilates. Despite the high search volume among individuals in their 40s, actual exercise habits are low among those in their 30s to 50s. Search volumes for instructor-led exercises are higher in cluster 1 than in other cluster areas, and the total number of searches decreases as the community size decreases. These results suggest that trends in search behavior depending on sex, age, and environment factors are essential when prescribing exercise for effective behavioral change.

## Introduction

Exercise has shown beneficial effects in various conditions, such as cardiovascular diseases, weight loss, and cancer [1]. However, the lack of physical activity is the fourth most common risk factor for mortality after hypertension, smoking, and hyperglycemia [2]. It is also a major social problem in Japan, and the Japanese Sports Agency has set a target of having 70% of adults aged >20 years exercising at least once a week in the 3rd Basic Plan for Sports [3]. Despite this, a 2022 survey found that only 52.3% of adults meet this goal, with lower participation rates particularly among those aged 20-50 years [4].

Although exercise prescription by physicians for individuals is beneficial in initiating or intensifying physical activity [5], providing specific exercise prescriptions is challenging for physicians; therefore, exercise is not often prescribed [6,7].

Exercise adherence involves various factors, including participant characteristics, knowledge of the benefits of exercise, enjoyment, social support, communication, and self-efficacy [8-10]. Immediate rewards, such as enjoyment, are especially important for maintaining exercise routines [11-15].

Therefore, to encourage patients to continue exercising, understanding trends in exercise preferences based on patient background may make it easier for doctors to prescribe exercise.

In this study, we used data from internet search keywords to compare the search volume of exercise-related keywords based on sex, age, and prefecture, as there is no existing research investigating interest in physical activity using internet search keywords. This study aimed to understand trends in exercise-related internet search keywords based on sex, age, and environmental factors in order to help doctors prescribe exercise more easily.

## Methods

### Data Sources

To examine the pattern of internet search interests in exercise, we used the search volume for a certain period extracted from Yahoo! JAPAN. Google is the most commonly used search engine in Japan, but its usage rate in Japan is lower than that in other countries. The search engine market shares in 2022 were 15% for Yahoo! JAPAN and 76% for Google [16]. Search volume data were retrieved with authorized access from the Yahoo! JAPAN Corporation server via the DS.INSIGHT People portal. Yahoo! JAPAN DS.INSIGHT is a valuable research tool that analyzes Yahoo! JAPAN’s big behavioral data, such as keywords, period, sex, age, and prefecture, and the transitions and trends in these searches over time [17,18]. The internet user population is the number of internet users throughout Japan, calculated based on the *Telecommunications Usage Trends Survey* published by the Ministry of Internal Affairs and Communications.

### Search Queries Used in the Analysis

To investigate the trend of internet searches on exercise, we conducted an observational study using the annual search volume of query terms obtained from Yahoo! JAPAN.

The search terms “ピラティス” (ie, “Pilates” in Japanese), “ラジオ体操” (ie, “radio calisthenics” in Japanese), “ストレッチ” (ie, “stretch” in Japanese), “ヨガ” (ie, “yoga” in Japanese), “筋トレ” (ie, “muscle training” in Japanese), “エアロバイク” (ie, “exercise bike” in Japanese), “ウォーキング” (ie, “walking” in Japanese), “太極拳” (ie, “tai chi” in Japanese), “ランニング” (ie, “running” in Japanese), and “登山” (ie, “hiking” in Japanese) were used to assess internet search interest in exercise from January to December 2022 (Multimedia Appendix 1).

Ten terms were selected in descending order of search volume, excluding competitive sports, from those listed in the exercise metabolic equivalent of task table published by the Ministry of Health, Labour and Welfare [19] (Multimedia Appendix 2). Competitive sports were excluded because of the anticipated increase in search volume attributed to these activities, such as watching professional sports and searching for game results.

This study considered the total search volume for each term, total search volume and percentage based on sex, search percentages based on age group, and characteristic score based on prefecture. The characteristic score is a proprietary aspect of Yahoo! JAPAN, and the specific analysis method has not been explicitly disclosed.

However, it is understood that the characteristic score indicates how uniquely a term is searched for in each prefecture. A baseline score of 2.5 suggests that the term is equally searched across all prefectures. Scores range from 0 to 5 in 0.5-point increments, with higher scores suggesting that the term is more distinctively searched for in that prefecture, regardless of overall search volume. The scores are normalized to account for differences in population size and age distribution across prefectures, ensuring that demographic variations do not bias the results.

### Statistical Analyses

Hierarchical cluster analysis (Ward method) was performed using the characteristic score of the 10 terms for each prefecture as variables. A dendrogram was created from this analysis, and 4 clusters were selected.

To understand if there are differences in population size and industries among the 4 clusters, data were collected for each prefecture from the Japanese government’s statistical portal site, e-Stat [20]. The collected data included the total population, population in densely inhabited districts (DIDs), percentage of population engaged in the primary industry, percentage of population engaged in the secondary industry, and percentage of population engaged in the tertiary industry for each prefecture.

In Japan, a DID is defined as a basic unit area with a population density of ≥4000 individuals per square kilometer. These areas are located within the boundaries of adjacent cities, towns, or villages, and the combined population of these adjacent areas exceeds 5000 during the national census [21].

Kruskal-Wallis tests, including Bonferroni correction for multiple comparisons, were conducted to analyze the differences among the 4 clusters using these collected data. The objective of this analysis was to determine the regional characteristics among the clusters formed through hierarchical cluster classification.

All statistical analyses were performed using IBM SPSS Statistics (version 27.0), with a significance level set at *P*<.05, following Bonferroni correction.

### Ethical Considerations

This study adhered to the ethical guidelines for Medical and Health Research Involving Human Subjects of Japan’s Ministry of Health, Labour and Welfare [22]. In accordance with this guideline, as this study used previously anonymized and deidentified data, an ethical review was waived, and patient informed consent was not required. This study conformed to the ethical principles of the Declaration of Helsinki (Fortaleza Revision, 2013) [23].

## Results

In 2022, the top searched term was “Pilates,” with 266,000 queries. The least searched term was “hiking,” with 40,400 queries (Table 1).

**Table 1:** Search patterns for exercise-related terms, based on total volume, sex, and age.

Parameter		Pilates	Radio calisthenics	Stretch	Yoga	Muscle training	Exercise bike	Walking	Tai chi	Running	Hiking	Internet user
**Search volume**												
	Total, n	266,000	134,000	120,000	117,000	111,000	99,600	66,900	65,900	40,700	40,400	99,499,669
	Male individuals, n (%)	66,900 (25.2)	47,900 (35.7)	54,800 (45.7)	30,600 (26.2)	65,800 (59.3)	48,400 (48.6)	31,000 (46.3)	20,600 (31.3)	25,400 (62.4)	23,400 (57.9)	50,286,908 (50.5)
	Female individuals, n (%)	199,000 (74.8)	85,900 (64.1)	65,600 (54.7)	86,200 (73.7)	45,100 (40.6)	51,200 (51.4)	35,900 (53.7)	45,300 (68.7)	15,300 (37.6)	17,000 (42.1)	49,212,761 (49.5)
**Search volume by male individuals according to age group (years; %)**												
	<20	5	5	7	5	13	8	5	5	8	3	14
	20s	17	5	6	8	14	12	6	6	11	8	13
	30s	18	12	11	15	19	19	13	8	18	14	14
	40s	21	21	21	24	25	25	22	14	25	22	17
	50s	17	16	22	21	16	17	20	15	21	22	16
	60s	13	17	18	13	8	11	20	18	11	18	13
	≥70	9	24	15	14	5	8	14	34	6	13	13
**Search volume by female individuals according to age group (years; %)**												
	<20	6	3	5	3	9	6	4	4	9	2	14
	20s	18	4	6	8	10	13	9	4	13	8	12
	30s	17	16	12	16	15	18	15	6	18	14	14
	40s	23	27	26	31	27	25	26	15	25	25	17
	50s	19	18	23	23	20	20	21	20	19	23	17
	60s	11	15	17	11	12	12	16	23	11	16	13
	≥70	6	17	11	8	7	6	9	28	5	12	13

The search percentages for “running” (25,400/40,700, 62.4%), “muscle training” (65,800/111,000, 59.3%), and “hiking” (23,400/40,400, 57.9%) were higher for male individuals than female individuals. However, female individuals showed higher search percentages than male individuals in the categories of “Pilates” (199,000/266,000, 74.8%), “yoga” (86,200/117,000, 73.7%), and “tai chi” (45,300/65,900, 68.7%; Table 1).

For male individuals, the search percentages across different age groups were highest for “Pilates,” “yoga,” “muscle training,” “exercise bike,” “walking,” “running,” and “hiking” in the 40-49 years age group. “Stretch” showed the highest search percentage in the 50-59 years group, whereas “tai chi” and “radio calisthenics” were the highest in the 70-79 years group.

For female individuals, the search percentage for “tai chi” was the highest in the 70-79 years age group; for the other 9 terms, the highest search percentages were observed in the 40-49 years age group (Table 1).

**Figure 1:**
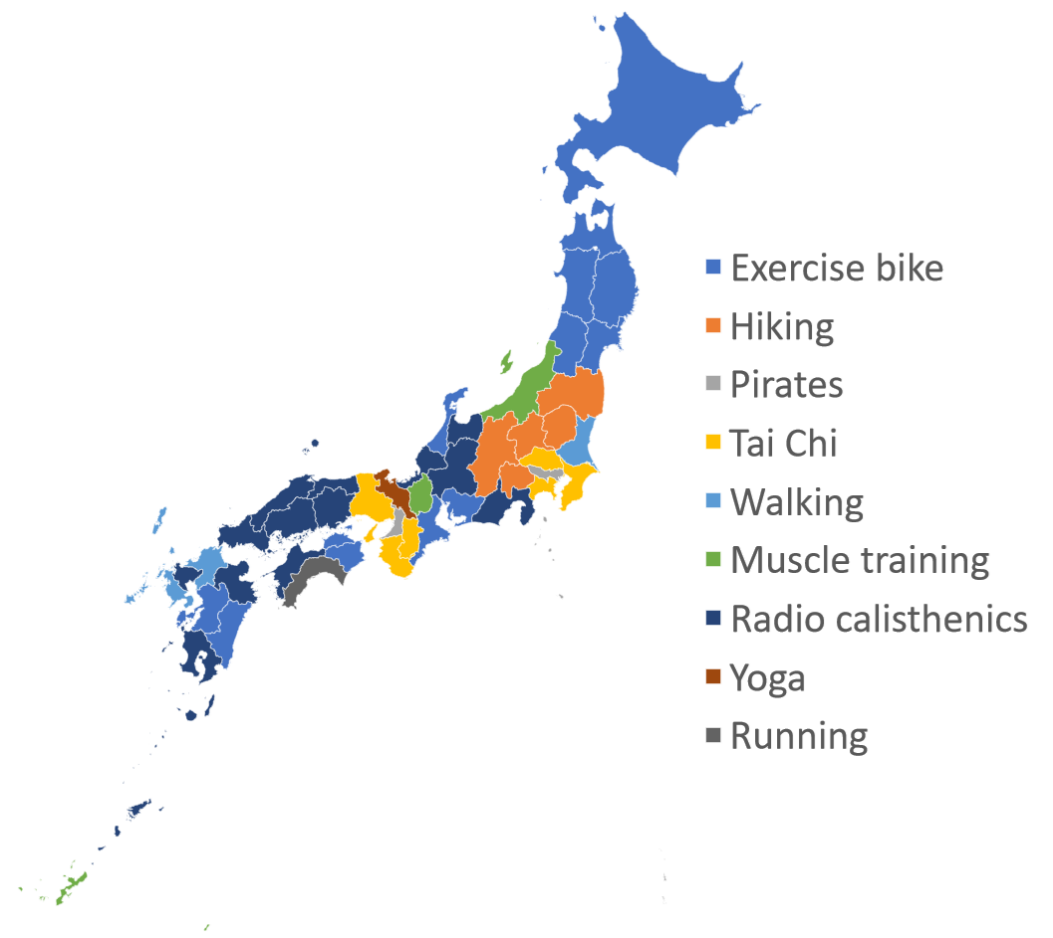
The term with the highest characteristic score for each prefecture.

Figure 1 shows the most distinct terms in each prefecture. The results suggest general trends, such as a higher likelihood of searching for “exercise bike” in cold snowy regions in the north; “hiking” in mountainous areas, such as Koshin; “Pilates” and “tai chi” in urban centers (around Tokyo and Osaka); and “radio calisthenics” in rural regions, such as Chugoku.

**Figure 2:**
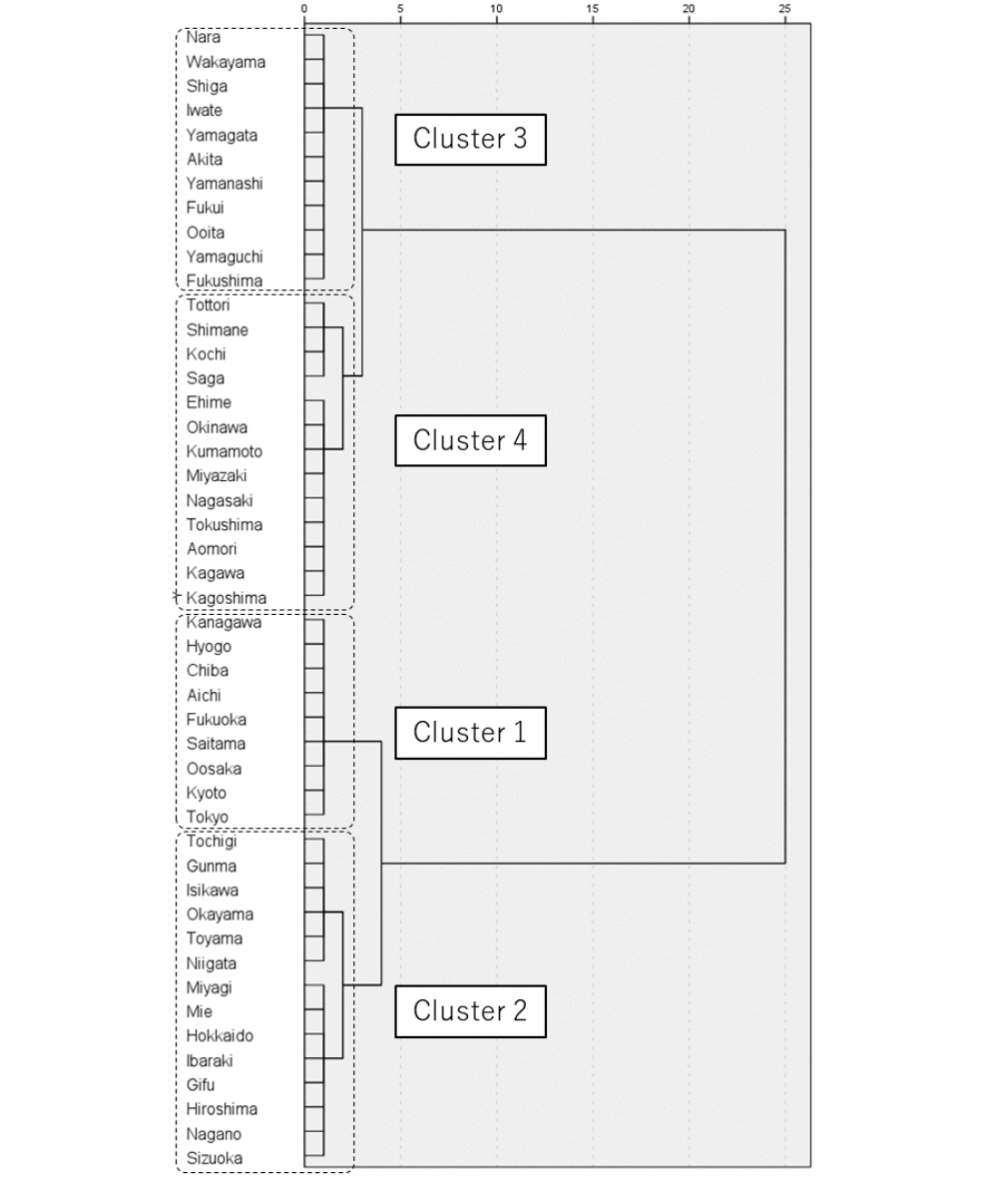
Classification of prefectures based on characteristic scores of exercise-related search terms, using hierarchical cluster analysis.

Figure 2 depicts the dendrogram resulting from hierarchical cluster analysis using the characteristic scores of each term across all 47 prefectures as variables. The dendrogram reveals 4 distinct clusters. Cluster 1 comprises 9 prefectures, including Tokyo, Aichi, Osaka, and Fukuoka, which are considered urban areas and encompass Japan’s 4 major cities. Cluster 2 is composed of 14 prefectures, including those situated in the central part of Honshu and excluding metropolitan and regional hub cities, such as Hokkaido, Miyagi, and Hiroshima. Cluster 3 encompasses 11 prefectures, mainly the suburbs of the Kinki region (Nara, Wakayama, and Shiga) and the Tohoku region (Yamagata and Akita) on the main island of Honshu. Cluster 4 includes 13 prefectures referred to as rural areas, such as Shimane and Tottori, at the periphery of Honshu, and regions in Kyushu and Shikoku.

Table 2 presents the average characteristic scores for each cluster. Cluster 1 had the highest scores for Pilates, stretch, yoga, and tai chi. Cluster 2 had the highest scores for radio calisthenics, muscle training, exercise bike, walking, and hiking. Cluster 4 had the lowest scores across all characteristics.

**Table 2:** Average characteristic scores by cluster.

Cluster	Pilates	Radio calisthenics	Stretch	Yoga	Muscle training	Exercise bike	Walking	Tai chi	Running	Hiking
Cluster 1, mean (SD)	3.22 (0.25)	2.61 (0.31)	2.89 (0.21)	2.94 (0.16)	2.72 (0.25)	2.50 (0.47)	2.61 (0.31)	3.06 (0.50)	2.61 (0.21)	2.72 (0.25)
Cluster 2, mean (SD)	1.93 (0.42)	3.00 (0.53)	2.43 (0.37)	2.32 (0.45)	2.79 (0.41)	3.04 (0.30)	2.64 (0.35)	2.36 (0.40)	2.61 (0.43)	2.86 (0.55)
Cluster 3, mean (SD)	1.23 (0.49)	2.18 (0.32)	1.64 (0.31)	1.64 (0.22)	1.68 (0.32)	2.09 (0.29)	1.82 (0.39)	1.82 (0.39)	1.73 (0.25)	2.14 (0.37)
Cluster 4, mean (SD)	0.88 (0.29)	1.81 (0.46)	1.38 (0.35)	1.31 (0.31)	1.58 (0.51)	1.96 (0.54)	1.69 (0.42)	1.08 (0.38)	1.54 (0.41)	1.04 (0.31)

**Figure 3:**
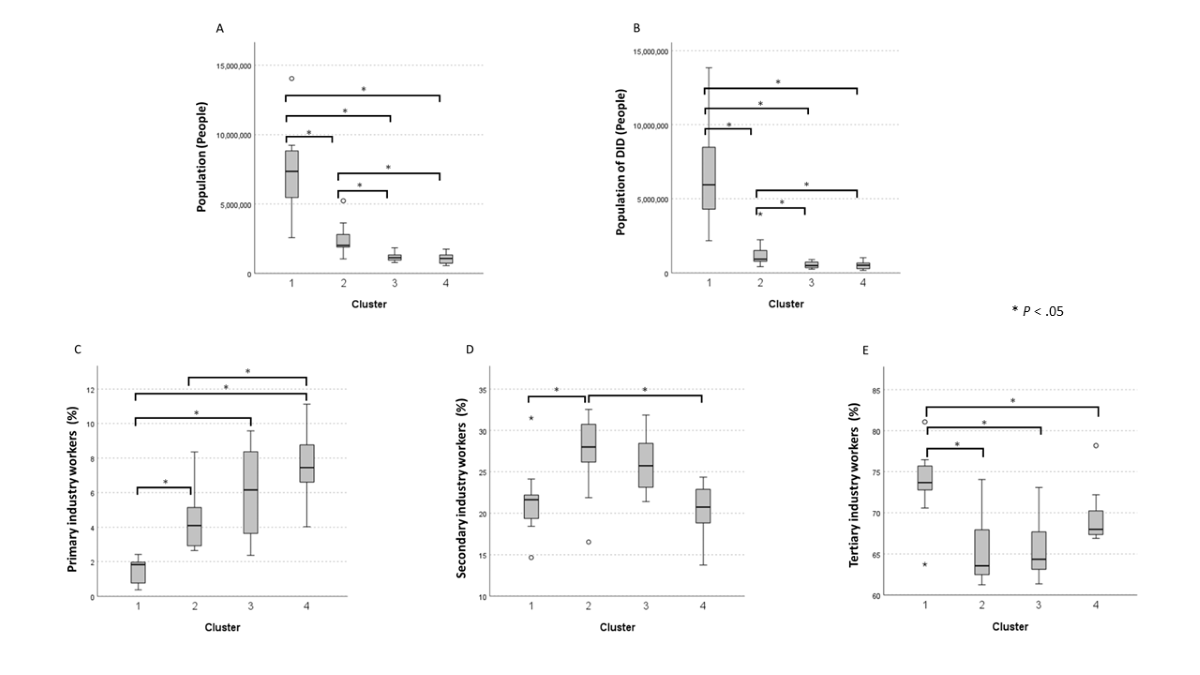
Demographic and occupational attributes of prefecture clusters: (A) population size, (B) population of DIDs, (C) primary industry workers, (D) secondary industry workers, and (E) tertiary industry workers. DID: densely inhabited district.

Subsequently, Kruskal-Wallis tests were conducted to examine whether there were any distinctive characteristics in population size and industries among the 4 clusters. The results are shown in Figure 3. The total and DID populations were higher in cluster 1, followed by cluster 2, with no significant differences observed between clusters 3 and 4 (*P*>.99). Although there was no significant difference in the percentage of primary industry workers between clusters 3 and 4 (*P*>.99) and between clusters 2 and 3 (*P*=.99), cluster 4 showed a tendency toward higher values than the other clusters. The percentage of secondary industry workers tended to be higher in cluster 2 than in other clusters. The percentage of tertiary industry workers was significantly higher in cluster 1 than in other clusters (all *P*<.05).

Based on these findings, cluster 1 was characterized as having a dominant tertiary industry and an overwhelming population size. Cluster 2 had a prevalent secondary industry and substantial population size and density. Clusters 3 and 4 were small and similar in population size; however, cluster 3 tended to have a larger secondary industry, whereas cluster 4 tended to have a larger primary industry.

## Discussion

### Principal Findings

This study revealed trends in search behavior depending on sex, age, and environment factors. The most frequently searched term was “Pilates,” a body-conditioning method that emphasizes the integration of the body, mind, and spirit, which was devised by Joseph Hubertus Pilates approximately 100 years ago (Table 1). This method has been particularly effective in reducing low back pain and associated disabilities [24]. In Japan, Pilates and yoga are often associated with relaxation and achieving a healthy physique, typically conducted by community instructors.

Previous studies have observed that male individuals seek improvement and a sense of achievement through exercise. In contrast, female individuals seek health, enjoyment, and community [25]. Consistent with prior studies, in this study, activities that offered improvement and a sense of achievement, such as muscle training, running, and hiking, had higher search percentages among male individuals than female individuals. Conversely, activities that promote health and can be performed within a community, such as Pilates, radio calisthenics, stretching, yoga, walking, and tai chi, had higher search percentages among female individuals than male individuals (Table 1). These findings support the results of previous studies and underscore the importance of considering potential sex-specific preferences when physicians prescribe exercise.

As people age, the preference for exercising with others increases, and the exercise purposes of male and female individuals become more similar [25,26]. In terms of age groups, it was generally observed that both male and female individuals had higher search percentages in their 40s (Table 1). However, surveys in Japan reveal that the age group engaging in the most exercise is those in their 60s, and the exercise participation rate is lower for individuals in their 30s to 50s [4]. This suggests that those in their 40s may not translate this interest into actual physical activity despite searching for exercise-related information. Physicians should encourage exercise and establish societal environments to ensure that individuals in their 40s, who are in this critical phase of interest, do not miss opportunities to engage in regular physical activity. In fact, initiating exercise even in middle and older age yields similar benefits in reducing the overall mortality risk compared with those who have been exercising since a younger age [27], underscoring the significant advantages of starting exercise in one’s 40s.

Regarding regional differences, a general trend emerged from the maps of characteristic scores based on prefecture, indicating that the choice to search for activities, such as hiking or using an exercise bike, is influenced by the proximity of natural environments and weather conditions (Figure 1). However, actual living environments within a prefecture, including urban and rural areas, vary. It can be challenging to fully capture the reality based solely on the characteristics of a prefecture. Therefore, we observed trends based on actual living and occupational environments by classifying the data into 4 clusters. This classification does not necessarily represent the characteristics of each prefecture. Instead, it reflects the diverse environments in which individuals live, even within a single prefecture, ranging from metropolitan to rural settings.

In cluster 1 areas, which have well-established social infrastructure, there was a higher frequency of searches for activities, such as Pilates, yoga, and tai chi, that require instructors (Table 2). In cluster 2 areas, where the environment lacks instructors for activities, such as Pilates and yoga, compared with cluster 1 areas, there is a tendency for searches related to accessible options, such as walking and radio calisthenics. The presence of a relatively larger community is suggested to positively affect the acquisition of exercise habits [28], potentially contributing to higher search volumes in both clusters 1 and 2.

**Figure 4:**
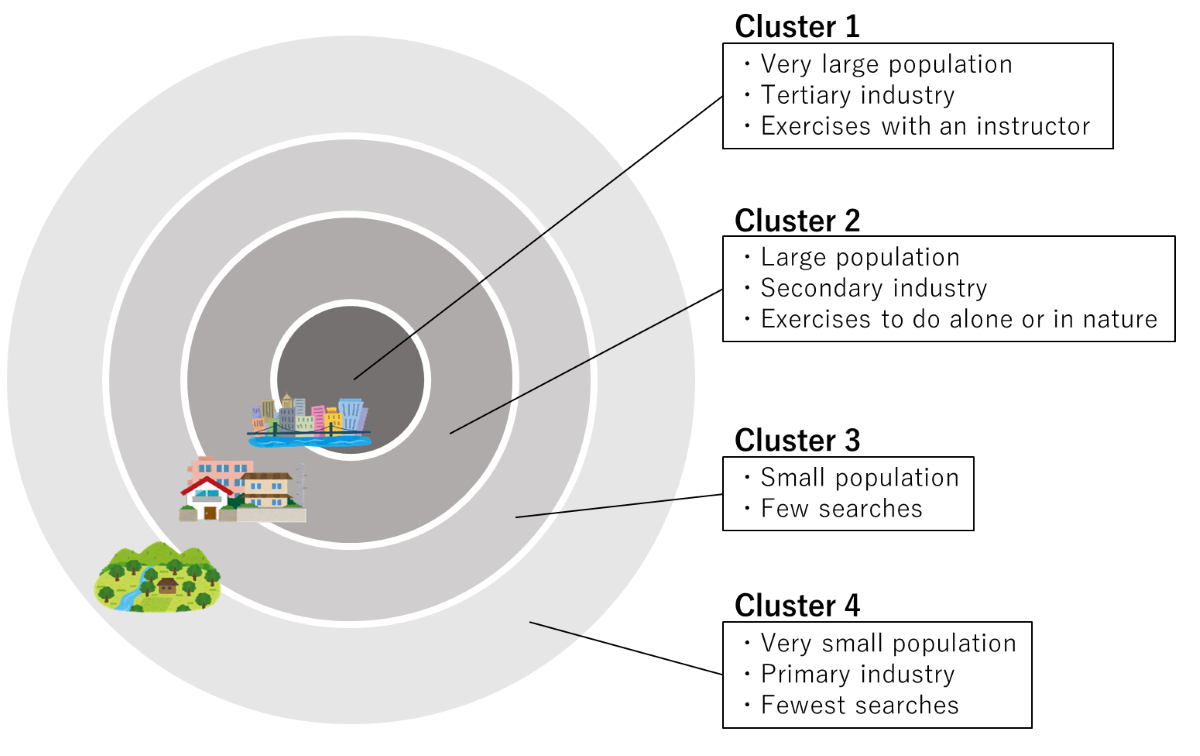
Regional variations in exercise preferences across clustered areas.

In cluster 3 areas, which are characterized by smaller communities, a limited community size is associated with a potential disadvantage in acquiring exercise habits [28], which may contribute to an overall lower search volume. In cluster 4 areas, it is assumed that primary industries dominate the area. Previous studies have suggested that in rural areas, the lack of infrastructure may limit exercise options to simple activities, such as walking or running, potentially diminishing interest in physical activity [29]. However, individual job characteristics must be assessed in order to obtain accurate information. Individuals engaged in primary industries may exhibit higher non–exercise-activity thermogenesis, indicating their potential to maintain a certain level of physical activity [30]. Figure 4 shows that even within a single prefecture or municipality, there are differences in exercise preferences depending on the surrounding population, urbanization, and industry.

In addition, there may be regional disparities in sex-related issues, such as masculinity and femininity, in Japanese society. The participation rate of male individuals in yoga is higher in urban areas than rural areas [31]. Society needs to create an environment that facilitates easy participation for male individuals.

In several foreign countries, approximately 70% of adults are reported to visit primary care outpatient services at least once a year [32]. From these data, it can be inferred that Japanese physicians likely have ample opportunities to prescribe exercise in outpatient settings. The “FITT-VP” method—an approach encompassing Frequency, Intensity, Time, Type, Volume, and Progression—and treating physical activity as a vital sign are recommended for assessing and prescribing exercise [1,33].

However, considering the challenges physicians face in prescribing exercise, uncertainties and difficulties in providing specific exercise prescriptions, along with time constraints, have become apparent. To address these challenges, the results of this study might serve as a valuable reference for tailoring exercise prescriptions to individual preferences based on patient backgrounds, such as sex, age, and environment factors.

### Limitations

To begin with, this study primarily reflects on search behaviors with unclear intentions. Although search terms for places to exercise increase the number of searches, exercises that can be performed without searching may be underestimated. Nevertheless, it is unlikely that the intentions behind these searches vary significantly across sex, age, and environment factors, which supports the validity of capturing search trends.

The search query count is linked to the prefecture where the search was made. For instance, the term “hiking” might be overrepresented in a prefecture if it was searched for by people who visit that prefecture. However, the impact is likely to be minimal given that people typically search “hiking” in the prefecture before visiting.

Japan has 4 seasons, which may cause fluctuations in search queries. We analyzed data over a full year to average out these seasonal effects. However, in practice, physical activity prescriptions should consider seasonal variations, as outdoor activities tend to increase in warmer months, while indoor activities like Pilates may remain consistent year round.

The cluster classification was based only on prefectures and was not observed within prefectures; therefore, it is impossible to understand the detailed regional characteristics. However, classifying them into 4 clusters allows for a better understanding of the medical service area, making it easier to prescribe exercises.

Due to the nature of this study, which relies on internet data, there are limitations concerning data from older age groups. When comparing demographic data from e-Stat with the proportion of internet users, the percentage of teenagers is about 2% higher in the general population. For each age group between 20 and 60 years, the percentage of internet users is approximately 2% higher than the general population.

However, for those in their 70s and older, while they account for 20% of male individuals and 26% of female individuals in the population, only 13% of both male and female individuals in this age group are internet users. Therefore, the study may not fully capture the interest in exercise of older adults in Japan.

The search terms for this study were selected based on our own considerations, which may introduce selection bias. The search term “ダンス” (ie, “dance” in Japanese) was considered for inclusion. Despite its high search volume of 77,400 queries, dance encompasses a wide range of activities, each with distinct characteristics. For instance, when specific types of dance, such as ballroom dance, belly dance, hula dance, or breakdance, are searched, the overall search volume decreases, and the distribution by sex and age varies. This variability in both search volume and demographic patterns, along with the fact that “dance” does not refer to a single, clearly defined type of physical activity, led us to exclude it from this study.

### Conclusions

Male individuals show more interest in individual activities, such as running, whereas female individuals are more inclined to search for group activities, such as Pilates. Despite the high search volume among individuals in their 40s, actual exercise habits are low among those in their 30s to 50s. Search volumes for instructor-led exercises are higher in regions characterized by tertiary industries and large population sizes (cluster 1), but the total number of searches decreases as the community size decreases. These results suggest that trends in search behavior depending on sex, age, and environment factors are essential for physicians when prescribing exercises for effective behavioral change.

## Data Availability

The datasets generated or analyzed during this study are not publicly available, complying with the Yahoo! JAPAN Corporation regulation. Still, they are available from the corresponding author upon reasonable request.

## Appendix

Multimedia Appendix 1 Description of each exercise.

Multimedia Appendix 2 List of competitive sports excluded from the search data.

## Abbreviations

DID: densely inhabited district
