# Supplementary material for: Trends in Exercise-Related Internet Search Keywords by Sex, Age, and Lifestyle: Infodemiological Study
Source: JMIR Form Res. 2024 Nov 11;8:e59395. doi: 10.2196/59395 (PMC11589504; doi:10.2196/59395)
Supplement: Multimedia Appendix 1 [file formative_v8i1e59395_app1.docx]

Multimedia Appendix 1. Description of each exercise

Pilates: Exercise focusing on core strength and flexibility that aims to improve balance, posture, and overall harmony of the body

・Radio calisthenics: Traditional Japanese exercise for all ages, combining stretching and strength training to music

・Stretch: Routine to improve flexibility and prevent muscle stiffness

・Yoga: Ancient practice that unifies mind and body through poses, breathing, and meditation, enhances flexibility and strength, and reduces stress

・Muscle training: Resistance exercises to build and strengthen muscles

・Exercise bike: Aerobic exercise using a stationary bike for cardiovascular benefits

・Walking: Low-impact aerobic exercise for overall fitness

・Tai chi: Traditional Chinese martial art with gentle movements and breathing techniques, promoting physical health and mental tranquility

・Running: High-impact cardiovascular exercise involving sustained jogging or sprinting

・Hiking: Outdoor activity involving walking in natural environments, often on trails
