# Supplementary material for: Trends in Exercise-Related Internet Search Keywords by Sex, Age, and Lifestyle: Infodemiological Study
Source: JMIR Form Res. 2024 Nov 11;8:e59395. doi: 10.2196/59395 (PMC11589504; doi:10.2196/59395)
Supplement: Multimedia Appendix 2 [file formative_v8i1e59395_app2.docx]

Multimedia Appendix 2. List of excluded competitive sports from search data

| Volleyball | Golf | Table tennis | Tennis | Baseball |
| --- | --- | --- | --- | --- |
| Basketball | Badminton | Soccer | Handball | Rugby |
